# Validating Indigenous Versions of the South African Personality Inventory

**DOI:** 10.3389/fpsyg.2021.556565

**Published:** 2021-05-20

**Authors:** Carin Hill, Mpho Hlahleni, Lebogang Legodi

**Affiliations:** Department of Industrial Psychology and People Management, College of Business and Economics, University of Johannesburg, Johannesburg, South Africa

**Keywords:** South African Personality Inventory, personality, indigenous, translations, Tshivenda, Sesotho, Exploratory Structural Equation Modelling

## Abstract

Personality assessments are frequently used to make decisions and predictions, creating a demand for assessments that are non-discriminatory. South African legislation requires psychological tests to be scientifically proven to be valid, reliable, fair and non-biased. In response to the necessity for a measure sensitive to indigenous differences, South African and Dutch researchers developed the South African Personality Inventory (SAPI). The SAPI represents a theoretical model of personality that uses an indigenous (emic) and universal (etic) approach to capture South Africa’s rich multicultural and multilingual view of personhood. The development of SAPI items and its simultaneous translation from English into all official languages necessitated the investigation of all the translated language versions’ psychometric properties. This study used Exploratory Structural Equation Modelling to examine the factor structure and model fit of two indigenous language versions of the SAPI, targeting the Tshivenda and the Southern Sotho languages. To accomplish this objective, Study 1 (*N* = 290) was done in South Africa among the Tshivenda ethnic group, while Study 2 (*N* = 293) was conducted in South Africa among the Sesotho ethnic group. An acquiescence response pattern was noticed in both studies, possibly to adhere to group consensus and emphasizing harmony within relationships. The ESEM solutions generated an excellent fit for both language versions, and most facets loaded acceptably on their expected factors. The Neuroticism factor proved to be problematic in both language versions. Within the Tshivenda version, the Emotional Stability facet did not generate adequate loadings on any SAPI factors. In contrast, neither Emotional Stability nor Negative Emotionality loaded sufficiently on the Neuroticism factor for the Southern Sotho version. While the overall fit of the six-factor model was excellent, the language in which a person completes a personality questionnaire seems to influence such an assessment’s factor structure. The Tshivenda and Sesotho versions of the SAPI cannot yet be positioned as equitable alternatives when using an indigenous version of the SAPI is needed. The implications of the results and proposals for future studies are discussed.

## Introduction

In van de Vijver and Rothmann (2004) anticipated that people from various ethnic groups would increasingly request psychological practices to be culture-specific and culturally informed. This projection came to pass within the South African context in which, over the last decade, psychological assessment has been considered problematic, given the general usage of western models and theories to measure and explain psychological constructs such as personality (Fetvadjiev et al., 2015).

The promulgation of the South African Employment Equity Act (EEA) 55 of 1998, Section 8 (Republic of South Africa, 1998), placed a spotlight on the cultural suitability of psychological tests and their usage within South Africa. The act explicitly states that all psychological measures used in South Africa should promote equality, be bias-free for all ethnic groups involved, and be scientifically valid and reliable (Republic of South Africa, 1998). Therefore, to adhere to the requirements of the EEA, it is necessary to create psychological measures, such as personality assessments, that are sensitive to ethnic differences. One way to address the requirements of the EEA is to develop personality assessments in all significant languages within a particular context (for example, South Africa) so that test-takers within that context understand the various personality trait definitions, and there is no discrimination against any language group (McCrae and Costa, 1997).

As in South Africa, there has also been a drive worldwide towards a more inclusive and integrated investigation and uncovering of more culturally specific personality taxonomies and assessing their prevalence across various cultural contexts. Cheung et al. (2011) highlighted in the need to integrate emic and etic approaches to study personality across cultures; thereby, culture-inclusive personality models can be unearthed, expanding the scope of mainstream personality psychology. An emic-etic approach to personality model development originates within a particular cultural context, expanding into comparisons with universal personality models (Cheung et al., 2011; Fetvadjiev et al., 2021). The emic approach aims to determine to what extent personality constructs are unique to specific cultures (Rolland, 2002), whereas the etic approach examines behaviour across many cultures, determining the universality and replicability of a theoretical personality model (Berry, 1989; Rolland, 2002). A review of the literature indicates that the emic-etic approach is being applied more frequently in researching personality measurement (e.g., Govia and Paisley-Clare, 2013; Fetvadjiev et al., 2015; Ion et al., 2016; Zeinoun et al., 2017; Burtǎverde et al., 2018; Burtaverde et al., 2019).

Given the concerns regarding psychological assessments specifically within the South African context, the South African Personality Inventory (SAPI) project was initiated, and it aimed to adhere to local legislation and develop a theoretical framework and personality measure that can be administered validly and reliably in all 11 official languages (Hill et al., 2013). The project developed a comprehensive personality assessment in a non-Western culture and serves as an example of a research program that used the emic-etic approach to instrument development (Cheung et al., 2011). The SAPI development consisted of two phases: a qualitative phase that focused on developing a theoretical model and a quantitative phase aimed at scale development and validation (Fetvadjiev et al., 2015).

### Qualitative Phase

Cheung et al. (2011) noted that the various South African languages do not all “…have easily accessible dictionaries” (p. 599), and therefore the traditional lexical approach to personality assessment development was not suitable for the SAPI project. Thus, the qualitative stage used an indigenous approach to gather personality descriptors from the 11 language groups. An indigenous approach can be described as an integration of the emic-etic methodology. Personality descriptors for the various South African language groups were obtained through face-to-face interviews. During these interviews, participants were asked to describe themselves and other persons that they know well. These persons included a parent, a grandparent, an eldest child/brother/sister, friends of the same and opposite sex, a colleague or friend from another ethnic group, a person who is psychologically very different from the participant, as well as least and most favourite teachers (Nel, 2008). All interview responses were translated into English, and the etic-emic approach was used to guide the content analysis of the interview responses. Applying the emic approach, the content analyses uncovered traits that were unique to certain language groups (for example, “being consistent” was only found in Afrikaans), semi-common facets (for example, “being tolerant” did not emerge within the Sotho group of languages), and common to all groups (for example, “being approachable, caring, and friendly” were found in all language groups; Nel, 2008).

Nel et al. (2012) described how the etic approach was applied in the SAPI development by using models such as the Five-Factor Model (FFM) and the HEXACO model to partly inform grouping the subclusters into a theoretical model with nine personality factors. A review of prominent personality models helped group the facets into subclusters based on shared content and behavioural styles (Nel et al., 2012). These subclusters were given collective labels and categorized into nine overarching clusters: Conscientiousness, Emotional Stability, Extraversion, Facilitating, Integrity, Intellect, Openness, Relationship Harmony, and Soft-heartedness. Cheung et al. (2011) (p. 7) summarized the process as extracting over 50,000 expressions and reducing it “… to 550 subfacets, then to 191 facets, 37 subclusters, and 9 clusters.” Table 1 presents an example of the reduction process from utterance to cluster.

**TABLE 1:** Clusters, subclusters, facets of personality-descriptive terms, and example responses.

Cluster	Subcluster	Facet (number of languages where facet appears/number of response represented under facet)	Example Response (Language)
Relationship Harmony	Conflict Seeking	Argumentative (10/105)	Likes to quarrel (Xhosa)
		Provoking (5/59)	Provocative and calls people names (Swati)
		Troublesome (11/337)	Creates tension for nothing (Zulu)
Softheartedness	Amiability	Friendly (11/740)	She is a friendly person (Tsonga)
		Irritating (7/93)	He is annoying and irritating (S. Sotho)
		Kind (11/1288)	Kind (Venda)
		Likeable (10/183)	He is loved by everyone (S. Sotho)
		Pleasant (9/201)	He was a nice person to live with (Zulu)
		Stern (7/24)	Always serious, not smiling (Xhosa)

**Source.*Nel et al. (2012).*

The nine personality factors displayed similarities and differences compared to the Big Five, the HEXACO, and the Chinese Personality Assessment Inventory (CPAI; Nel et al., 2012). Their findings confirmed the universality of specific personality dimensions, but there was a difference in the representation of the “traditional” personality dimensions. For example, while Agreeableness and Extraversion are the Big Five’s largest dimensions, the SAPI produced more social-relational and agreeable characteristics than Extraversion, Conscientiousness, or Emotional Stability concepts. Nel et al. (2012) noted that the SAPI’s Integrity factor correlates with the HEXACO Honesty-Humility factor; however, the SAPI Integrity factor focuses more strongly on matters of fairness and discrimination rather than greed-avoidance and modesty. Lastly, the CPAI and the SAPI’s Interpersonal Relatedness dimensions differ specifically in that the Face-saving motives characteristic of the CPAI is not prominent in the South African context.

### Quantitative Phase

The quantitative stage of the SAPI related to item development and covered two multifaceted phases: First, the development and simultaneous translation of items, and then the empirical evaluation of said items. Items representing the theoretical nine SAPI clusters were developed through construct modelling, which involved 1) grouping the initial responses by facet and extracting content-representative responses; 2) describing and defining the nine SAPI facets by making use of content-representative responses; 3) converting qualitative responses generated from the interviews into item stems; 4) creating items from the item stems the SAPI team believed tapped the various facets; 5) pilot testing the items and 6) analyzing the gathered data to confirm the extent to which the results are similar to the primary plans as set out in the construct map (Hill et al., 2013).

The process of transforming item stems into items was guided by Hendriks et al. (1999) suggestions. For example, items needed to represent concrete behaviour, item formulations should not be too difficult to understand, excluding language-specific items and omitting negations. The items were formulated in the direction of the construct to exclude needless linguistic complexity and reduce possible construct irrelevant variance (Abedi, 2006). According to Suárez-Álvarez et al. (2018), combining regular and reversed items in an assessment causes measurement precision to be weak, jeopardizes unidimensionality due to secondary sources of variance, reduce the variance of the combined items, and the verbal skills of participants will influence their responses.

Ultimately a pool of 2,574 items was developed for the nine SAPI factors, and given the large number of items, each of the nine clusters’ items was evaluated in separate studies. The items were administered in English because it is frequently spoken and understood by all the ethnocultural groups in South Africa. Moreover, English was deemed a suitable choice as, according to (Fetvadjiev et al., 2015, p. 829), “English has one of the richest lexica for personality description.” The initial pool of 2,574 items was reduced to 571 items through an extensive analysis of the items that involved using psychometric and substantive criteria to remove or retain items. The psychometric criteria entailed using the results from the pilot studies to eliminate items that had unacceptable mean values, skewness, or kurtosis (Fetvadjiev et al., 2015). Items were also subjected to factor analysis, and subsequently, items with loadings of less than 0.30 on either the facet or cluster were removed. Substantive criteria included electing to include items that represented the constructs excellently, items that did not display great similarity within and across clusters, as well as items that “were most in line with the formulation rules of behaviour focus, simple language, and translatability” (Fetvadjiev et al., 2015, p. 829). Through these criteria, the pool of items was reduced to 571.

Another step in decreasing the number of items was translating the 571 items and eliminating items that were considered potentially challenging to comprehend. The 571 English items were translated through a rigorous process into the remaining ten official languages of South Africa. During this process, the SAPI was translated from English into the remaining ten official South African languages by professional language translators. Another group of independent translators with general knowledge regarding the psychological construct beings studied then checked the translations by translating the various language versions back to English. These translations were then discussed and debated during meetings between the SAPI team and the various expert translators. This approach allowed people from the relevant cultural and language groups with general knowledge regarding the psychological construct beings studied to influence the test development process by alerting the test developers to any language or cultural idiosyncrasies and safeguards against cultural and linguistic centring within items (Rogers et al., 2003; Tanzer, 2005). Thereby the constructs clarity is enhanced, and the test items are deemed more relevant and representative of the predefined culture or language groups (Tanzer, 2005). During the translation process, 181 items were possibly too difficult to understand, 100 were considered too complicated, and 40 items seemed to use abstract trait terms (Fetvadjiev et al., 2015).

Lastly, the remaining 250 items were administered to a diverse sample, utilizing factor and reliability analyses to determine and eliminate 104 items that decreased ideal factor replicability or that lowered the overall reliability of the facets (Hill et al., 2013; Fetvadjiev et al., 2015). Using factor analysis, the reduced item set of 146 items generated six empirical clusters as opposed to the initial nine theoretical clusters. The six clusters were: Conscientiousness, Extraversion, Neuroticism, Openness, Social-Relational Negative (SR-Negative), and Social-Relational Positive (SR-Positive) (Fetvadjiev et al., 2015). However, upon subsequent analyses, an additional 24 items were carefully chosen from the SAPI item repository and included in the questionnaire to reinforce the Conscientiousness, openness, and both the negative and positive social–relational factors (Morton et al., 2019). As it stands, the current English version of the instrument, the SAPI-EN, consists of 170 items, representing 20 facets and six factors. The definitions of the various factors and their associated facets can be found in Table 2.

**TABLE 2:** Definitions of SAPI factors and associated behaviours.

SAPI Factor	Definition	Typical Behaviours
Conscientiousness	To be determined, precise, thorough, organized, detail- and goal-focused, punctual, reliable, cautious, and emotionally controlled.	Doing a thorough job, being organized, showing concern, always being on time, and being accurate and persistent.
Extraversion	To be sociable and talkative, interacting with people in a spontaneous manner.	Being outgoing, sociable, enthusiastic, active and energetic.
Neuroticism	To be impulsive and to fluctuate between emotions.	Being nervous, anxious, easily upset or embarrassed, worried and afraid of being judged by others.
Openness	To be well-informed and observant of external and internal things, being a rational and progressive thinker, and acquiring new experiences, knowledge, skills, and ideas.	Being artistic, observant, having an active imagination, being open to new experiences, smart, intelligent and sophisticated.
SR-Negative	To approach relations with others controversially.	Being critical, tending to find fault with others, being quarrelsome and rude to others, and acting as if one is better than the others.
SR-Positive	To positively manage relations with others.	Guiding others, helping people to improve, making others feel comfortable, taking care not to hurt others, aiming at harmonious resolution of conflicts, being considerate, and taking others’ needs and feelings into account.

**Source*: SAPI Feedback Report (2020).*

The psychometric properties of the SAPI-EN have been investigated and validated in several studies within the South African context that looked at its factor structure (Nel et al., 2012; Fetvadjiev et al., 2015; Morton et al., 2019), measurement invariance (Fetvadjiev et al., 2021; Morton et al., 2020), and its predictive validity (Morton et al., 2018). Fetvadjiev et al. (2015) investigated the extent to which the SAPI link with the Big Five by conducting a joint factor analysis and regression analysis on a Big Five measurement’s facets and the SAPI facet scales. They found that two social-relational factors beyond the Big Five could be identified but that the Big Five did, to some extent, covary with the variance of the two social-relational factors (Fetvadjiev et al., 2015). The replicability of the SAPI’s six-factor structure among New Zealand European and Māori students were recently investigated, and the SAPI structure was found to be equivalent and had metric variance between the New Zealand European and Māori students (Fetvadjiev et al., 2021). These results revealed that an indigenously derived model such as the SAPI could be applicable outside of its country of origin (Fetvadjiev et al., 2021).

The next step in the process of refining the SAPI was to ensure that the various translations still measure the same constructs as the original English version. The language generally understood across the South African landscape and most often used within the business, political, and media spheres are English; however, administering a psychometric instrument in English may increase the likelihood of incorrect responses (van de Vijver and Rothmann, 2004; Meiring et al., 2006; Grobler, 2014). Many personality assessments are conducted in English, which may prove problematic since the study of literature by Gee et al. (2010) showed that poor English proficiency might restrict an individual’s employment opportunities and increase experiences of discrimination. It is, therefore, important that individuals are given the opportunity to complete personality assessments in a language with which they are comfortable, and this is often their home language (Gee et al., 2010).

South Africa has four main ethnic groups (Africans, Coloureds^1^, Indians, and Whites) and 11 official languages that consist of two West Germanic languages (English and Afrikaans) and nine Bantu languages. The nine Bantu languages can be separated into three categories: (1) three Sotho languages, which are sub-categorized into Setswana, Sepedi, and Sesotho; (2) four Nguni languages, sub-categorized into IsiZulu, IsiXhosa, IsiNdebele, and SiSwati; and (3) Xitsonga and Tshivenda, which are originally of Shona descent (Nel, 2008). The assortment of languages and cultures contribute to South Africa’s unique environment and therefore increases the complexity of personality assessment in South Africa (Grobler, 2014). Consequently, South African psychologists have to carefully approach the significant tasks to assess and compare individuals from any of the 11 official language groups and to ensure the most suitable personality assessment is used (Grobler, 2014).

Therefore, the general objective of the current study is to present the factor structures and model fit of two indigenous versions of the SAPI, namely, the Tshivenda (SAPI-VE) and the Southern Sotho (SAPI-SS) versions. The research methodology (i.e., measuring instruments, sampling, data collection, and statistical analysis procedures) was the same across both studies, and a description of these processes follows.

## Research Methodology

### Measuring Instruments

#### South African Personality Inventory (SAPI)

The original SAPI-EN^2^ (English version) is a 170-item scale measuring six factors with their accompanying facets: Conscientiousness (Achievement Oriented, Orderliness, Traditionalism-Religiosity), Extraversion (Playfulness, Sociability), Neuroticism (Emotional Balance, Negative Emotionality), Openness (Broadmindedness, Epistemic Curiosity, Intellect), SR-Negative (Arrogance, Conflict Seeking, Deceitfulness, Hostility/Egoism), and SR-Positive (Empathy, Facilitating, Integrity, Interrelatedness, Social Intelligence, Warm-Heartedness). The entire Emotional Balance facet was reversed scored in order to have the same direction as the Negative Emotionality facet. The scale uses a five-point Likert-type format ranging from 1 (strongly disagree) to 5 (strongly agree). Fetvadjiev et al. (2015) found acceptable Cronbach alpha coefficients for the SAPI-EN six-factor solution ranging between 0.61 and 0.86, while Morton et al. (2018) found reliabilities ranging between 0.61 and 0.88. No items were reversed scored.

For this study, the Tshivenda and Southern Sotho versions of the translated SAPI were again sent to independent language experts before its administration to verify its accuracy. This process ensured that the translation validity (i.e., content and face validity) of the SAPI-VE and SAPI-SS was established. Examples of paraphrased items for the various facets are presented in Table 3.

**TABLE 3:** Examples of paraphrased items for the different language versions.

Facet	English	Southern Sotho	Tshivenda
Achievement Orientation	I get motivated by my goals	Ke ipehela maikemisetso	Ndi a ^ divhetshela zwipikwa
Arrogance	I act arrogantly	Ke itshwere ka tsela e ikgantshang	Ndi ^ difara zwavhu^ di
Broad-Mindedness	I seek new experiences	Ke ikemiseditse ho leka dintho tse ntjha	Ndi funa u lingedza zwithu zwiswa
Conflict-Seeking	I cause fights	Ke qala le ho tena ba bang	Ndi a levhela vhaṅwe
Deceitfulness	I mislead others	Ke leka ho qhekanyetsa batho	Ndi a lingedza u fhura vhaṅwe
Emotional Balance	I calm down easily	Ke kgona ho imamela ke kgutse kapele	Ndi a tavhanya u dzika
Empathy	I consider others’ feelings	Ke kgona ho mamella maikutlo a ba bang	Ndi a dzhiela n^ tha vhu^ dipfi ha vhaṅwe vhathu
Epistemic Curiosity	I love learning more about the world	Ke batlisisa tse ngata ka lefatshe	Ndi ^ to^ desa u ^ divha nga ha shango
Facilitating	I give guidance to people in their life decisions	Ke fa ba bang keletso ka bokamoso ba bona	Ndi a ^ netshedza vhaṅwe ngeletshedzo nga ha vhumatshelo
Hostility–Egoism	I make people feel vulnerable	Ke etsa hore ba bang ba ikutlwe e le dithooto	Ndi ita uri vhaṅwe vha pfe vha zwi^ dahela
Integrity	I acknowledge my mistakes	Ke a amohela ha ke fositse	Ndi a tenda arali ndo khakha
Intellect	I learn new things easily	Ke kgona ho ithuta kapelenyana	Ndi a kona u guda nga u ^ tavhanya
Interpersonal Relatedness	I help people live in peace	Ke thusa ba bang ho bopa kgotso mahareng a bona	Ndi thusa vhathu uri vha farelane
Negative Emotionality	I get angry a lot	Ke kgentshwa ke ditaba tse nyenyane	A thi lengi u sinyuwa
Orderliness	I do things with precision	Ke sebetsa ka ho hlaka mosebetsing wa ka	Ndi a dodombedza mushumo wanga
Playfulness	I enjoy laughing with others	Ke tshehisa ba bang	Ndi a vha seisa
Sociability	I chat with many people	Ke buisana le motho ofe kapa ofe	Ndi haseledza na vho^ the
Social Intelligence	I understand how people feel	Ke kgona ho utwlisisa maikutlo a ba bang	Ndi a kona u pfesesa vhu^ dipfi ha vhaṅwe vhathu
Traditionalism–Religiosity	I believe in tradition	Ke dumela ho dintho tsa bohlokwa tsa setho	Ndi tenda kha ndeme ya sialala
Warm-Heartedness	I support others when they need it	Ke thusa ba bang ha ba na le ditlhoko	Ndi a thusa vhaṅwe musi vhe na ^ tho^ dea

### Sampling

#### Sampling Procedures

A non-probability purposive sampling strategy was used to gather data, with the deliberate focus on either Tshivenda or Southern Sotho speakers. Participants were recruited during 2017 in and around the Gauteng province in South Africa in areas that are known to be mostly populated by either Tshivenda or Southern Sotho speaking people. Participation was voluntary, and no compensation was offered. Ethical clearance for the studies was given by the University of Johannesburg. Participants provided informed consent after the purpose and their rights were explained to them. The data is confidential and stored safely.

#### Sample Size

In both studies, using the confidence level was set at 95%, and the confidence interval was set at 5% to determine the required sample size. As per these calculations, a sample size of 384 was needed per study. However, a review of literature by Sakaluk and Short (2017) indicated that about 200 to 250 participants could be considered as an acceptable sample size. The missing values were classified as missing at random (MAR) and missing not at random (MNAR). All missing cases with more than 10% missing values were deleted. Missing values for cases with less than 10% values were replaced with the linear trend at that point; missing values are thus replaced with their predicted values, and this further assists with the interpretation of the data (Hinkle et al., 2003).

### Data Collection

Paper-and-pencil questionnaires were administered to the participants in the form of a booklet containing the questions and a separate answer sheet to record participants’ responses. To ensure that the participants were able to read either Tshivenda or Southern Sotho, they were asked verbally and on the questionnaire if they were proficient in reading the respective language.

### Statistical Analyses

SPSS Version 26 and Mplus 8.1 (Muthén and Muthén, 2018) were used to conduct all the analyses. The descriptive statistics (mean scores and standard deviation), Cronbach alphas, and amount of items per facet of the 20 facets were analyzed. Items that decreased the internal consistency of the facets were omitted from further analyses.

Marsh et al. (2013) noted that factor analysis had been the primary methodology to identify differences between individual, especially related to the commonly known five personality factors (Agreeableness, Conscientiousness, Extraversion, Neuroticism, and Openness). However, Marsh et al. (2013) determined from the literature that, while exploratory factor analysis (EFA) generally provides stable support for the five personality factors, confirmatory factor analysis (CFA) and structural equation modelling (SEM) are usually unsuccessful in generating acceptable fit. The lack of fit in this instance is largely due to the several cross-loadings associated with personality data, as well as CFA producing inaccurate factors when zero loadings have been misspecified (Asparouhov and Muthén, 2009; Marsh et al., 2013). Exploratory Structural Equation Modelling (ESEM) seems to be a solution to the various issues relating to factor analysis and personality measures since it is an overarching integration of the best aspects of traditional EFA and CFA (Asparouhov and Muthén, 2009; Marsh et al., 2009, 2013, 2014). According to (Marsh et al., 2011, p. 323), the ESEM methodology provides the researcher with access to typical CFA/SEM parameters and statistical advances: standard errors; goodness-of-fit statistics; comparisons of competing models through tests of statistical significance and fit indices; inclusion of correlated residuals; inclusion of both CFA and EFA factors based on the same, different, or overlapping sets of items; estimation of method effects and bifactor models; multiple-indicators multiple-causes models (MIMIC); multiple group and longitudinal invariance analyses; growth modelling.

Exploratory Structural Equation Modelling, therefore, may be a better analytical approach for personality researchers since it not only provides a better data fit but also generates more differentiated, thus less correlated, latent factors (Marsh et al., 2013). As such, the current study employed ESEM to analyze both the SAPI-VE and SAPI-SS datasets. The fit of the data when using ESEM to the hypothesized model was determined through firstly assessing the chi-square index (χ^2^). The χ^2^ measures the degree of difference between the data and the model, although a non-significant result is preferred since the χ^2^ is very sensitive to sample size (Wang and Wang, 2012). Additional model fit indices have also been evaluated. The model fit indices included the Comparative Fit Index (CFI), the Tucker-Lewis Index (TLI), the Root-Mean-Square Error of Approximation (RMSEA), and the standardized root mean square residual (SRMR) as executed in Mplus using the robust maximum likelihood (MLR) estimator (Muthén and Muthén, 2018). The rules of thumb to determine satisfactory fit, according to Hu and Bentler (1999), are >0.95 for both the CFI and TLI, RMSEA ≤ 0.06, and SRMR < 0.08.

As directed by Asparouhov and Muthén (2009), cross-loadings were permitted, and target rotation was chosen based on the *a priori* SAPI structure. The factor loading of each facet was inspected to determine whether the facet represented the intended factor. The guideline by Hair et al. (1998), which states that factor loadings need to be ±0.30, was used to evaluate the factor loadings. The strength of the correlation coefficients of the SAPI-VE/SAPI-SS factors was, respectively, judged according to Cohen’s (1988) criteria: >0.20 indicates a small effect; >0.30 indicates a medium effect, and >0.50 indicates a large effect.

### Ethical Approval

The research was approved by the University of Johannesburg Departmental Ethical Committee. Ethical clearance numbers: IPPM2017/061(M) and IPPM2017/101(M). The study involved human participants, and all procedures were in accordance with the ethical standards set out by the institutional research committee, as well as the Rules of Conduct Pertaining Specifically to Psychology as set out by the Health Professions Council of South Africa’s Professional Board of Psychology (Form 223).

#### Informed Consent

Informed consent was obtained from all participants included in the study.

## Study 1

Tshivenda is regarded as a minority language in South Africa since only 2.4% of the population speaks Tshivenda as a first language. Tshivenda is thus the second-least spoken language in the country (Statistics South Africa, 2011). Tshivenda is a relatively distinct language in its family of languages (Maree, 1998), although it does share linguistic features with Shona (a commonly used language in Zimbabwe) and Sotho (West and Morries, 1976). van Eeden and Mantsha (2007) 75 highlighted translation difficulties encountered when using the back-translation method to translate the Sixteen Personality Factor Questionnaire (16PF5) from English to Tshivenda: “[t]he level and understanding of the words being used, the understanding of the context and interrelationships of words, the understanding of phrases and idiomatic expressions, double meanings, and qualifying words could all have affected performance.” For example, there is no Tshivenda term for “depression” which made the literal translation of one of the items problematic; English idiomatic expressions could not be translated literally in Tshivenda; and, finally, the use of the negative form in Tshivenda confused test-takers (van Eeden and Mantsha, 2007). van Eeden and Mantsha (2007) study is the only one thus far that attempted to develop a Tshivenda-translated version of a personality questionnaire. They concluded that a literal translation from English to Tshivenda of a personality assessment would most likely be insufficient. The SAPI, therefore, heeded van Eeden and Mantsha’s call to use an indigenous approach to develop a personality test that can be used in African language groups, such as the Tshivenda people.

### Participants

#### Inclusion and Exclusion

This study focused specifically on the Tshivenda speaking people, 18 years and older, who understood Tshivenda and who could read the Tshivenda language.

#### Participant Characteristics

The sample consisted of 44% men and 54% women. The majority of the participants (48%) were within the age group of 18–27; 28% were between 28 and 37, and 15% were between 38 and 47. In terms of ethnicity, most of the participants identified as African (98%) and a small percentage (0.3%) identified as Coloured. Most of the participants (98%) spoke Tshivenda as a home language. Sixty-four percent of the participants had completed Grade 12 as their highest qualification, while 25% has completed a post-school qualification. Fifty-seven percent of the sample was able to read English, and only 1% of the sample rated their English reading ability as “very poor.”

#### Sample Size

After the deletion of cases with extreme missing values and multivariate outliers, the initial data set of 406 was reduced to 290.

### Results

The Tshivenda speaking participants (Table 4) indicated that they disagreed with all the items representing the perceived negative facets, as well as the Sociability facet. The participants were generally neutral in their responses towards the Negative Emotionality items while agreeing with all the perceived positive facets. No extreme response styles were frequently endorsed (for example, “strongly disagree,” “strongly agree”).

**TABLE 4:** Descriptive statistics of the SAPI-VE facets.

Variable	Mean	Std. Deviation	Alpha	Number of items
**Conscientiousness**				
Achievement Orientation	4.18	0.41	0.74	11
Orderliness	3.93	0.42	0.77	13
Traditionalism-Religiosity	3.99	0.64	0.44	3
Integrity	3.97	0.40	0.74	13
**Extraversion**				
Playfulness	3.91	0.50	0.57	6
Sociability	3.88	0.58	0.65	6
**Openness**				
Broad-Mindedness	4.08	0.46	0.64	6
Epistemic Curiosity	4.19	0.46	0.55	5
Intellect	3.86	0.42	0.70	10
**Neuroticism**				
Emotional Balance	3.97	0.45	0.61	7
Negative Emotionality	2.98	0.66	0.69	8
**SR-Negative**				
Arrogance	2.18	0.78	0.70	5
Conflict Seeking	2.15	0.73	0.65	5
Deceitfulness	2.35	0.69	0.71	7
Hostility Egoism	2.11	0.51	0.72	13
**SR-Positive**				
Integrity*	3.97	0.40	0.74	13
Empathy	4.08	0.45	0.63	7
Facilitation	4.00	0.44	0.78	10
Interpersonal Relatedness	4.05	0.43	0.71	8
Social Intelligence	4.21	0.49	0.66	4
Warm Heartedness	4.10	0.41	0.77	11

*The Integrity facet forms part of both the Conscientiousness and SR-Positive factors.*

A total of 12 items were removed since they decreased the facets’ reliability, and the facet displayed poor model fit. For more information regarding the item loadings, please see Appendix A. The Traditionalism-Religiosity facet retained only three items and displayed very poor reliability (α = 0.44); therefore, the model fit of the facet was evaluated. The model fit for the Traditionalism-Religiosity facet proved to be very poor, being just-identified with zero degrees of freedom and thus model fit could not be assessed (χ^2^ = 0.00; *df* = 0; CFI = 1.00; TLI = 1.00; RMSEA = 0.00; 90% CI for the RMSEA = 0.00-0.00; SRMR = 0.00). Given the aforementioned, the Traditionalism-Religiosity facet was removed from further analyses. The Cronbach alphas for the remaining 19 facets ranged between 0.55 and 0.78. Taking the research by Marsh et al. (2013) into account about the influence of the number of items on the reliability of personality factors, it seems to be reasonably acceptable to have a facet with five items and an internal consistency score of 0.55 (for example, Epistemic Curiosity).

The ESEM results produced excellent fit to the data (χ^2^ = 109.37; *df* = 72; CFI = 0.99; TLI = 0.97; RMSEA = 0.04; 90% CI for the RMSEA = 0.03-0.06; SRMR = 0.02). The results (Table 5) shows acceptable factor loadings ranging between 0.37 and 0.93 (*M* = 0.59) for most of the facets that loaded on their expected factor, except for Emotional Balance that did not generate any adequate factor loadings (λ ranged between −0.29 and 0.13). Cross-loadings were present in the ESEM model and, in some cases, may alter the definitions of the respective factors. Intellect loaded on both its expected factor, Openness (λ = 0.37), as well as Conscientiousness (λ = 0.39), while Empathy loaded on SR-Positive (λ = 0.30) and Extraversion (λ = 0.35). The differences in these cross-loadings were, however, marginal. Achievement Orientation generated a problematic cross-loading within the SAPI-VE, displaying a poor factor loading on its expected factor, Conscientiousness (λ = 0.25), but loading acceptably on the Openness factor (λ = 0.37). As such, the definitions of Conscientiousness and Openness may have to be altered. Integrity, as expected, loaded on both Conscientiousness and SR-Positive. Moderate to strong correlated factors emerged, especially between SR-Positive and Conscientiousness (*r* = 0.55) and SR-Positive and Openness (*r* = 0.67).

**TABLE 5:** Standardized estimates of the SAPI-VE based on the ESEM solution.

Variable	λ					
	C	E	O	N	SRN	SRP
**Conscientiousness**						
Achievement Orientation	0.25**	0.08	0.37**	–0.11	−0.13**	0.14*
Orderliness	0.52***	–0.06	0.19**	–0.12	–0.08	0.22
Integrity	0.41***	0.05	0.03	–0.04	−0.15***	0.45***
**Extraversion**						
Playfulness	0.09	0.55***	0.03	0.07	–0.01	0.16*
Sociability	−0.20***	0.61**	–0.02	−0.26***	0.11**	0.31**
**Openness**						
Broad-Mindedness	0.05	0.16**	0.78***	0.07	0.04	–0.07
Epistemic Curiosity	–0.11	−0.19**	0.85***	–0.02	−0.08*	0.11
Intellect	0.39***	0.08	0.37**	−0.27***	0.17**	0.11
**Neuroticism**						
*Emotional Balance*	−0.21**	–0.10	−0.29***	0.13	0.02	−0.24**
Negative Emotionality	–0.02	–0.14	0.00	0.65*	0.30	0.23**
**SR-Negative**						
Arrogance	0.04	−0.20**	0.00	0.07	0.61***	0.18
Conflict Seeking	–0.09	0.24***	0.05	0.10	0.64***	−0.19*
Deceitfulness	0.15	0.06	–0.01	0.15	0.59***	–0.21
Hostility Egoism	–0.09	0.03	0.05	0.00	0.93***	0.05
**SR-Positive**						
Integrity	0.41***	0.05	0.03	–0.04	−0.15***	0.45***
Empathy	0.30**	0.35***	0.17*	0.20	−0.22***	0.19
Facilitation	0.37***	0.10*	0.04	–0.09	–0.04	0.47***
Interpersonal relatedness	0.19*	0.10	0.05	–0.02	−0.11*	0.57***
Social Intelligence	−0.19*	0.15	0.29**	0.03	−0.13*	0.54**
Warm Heartedness	0.19*	0.22***	0.05	–0.01	–0.06	0.57***
**Factor correlations**						
Conscientiousness	1.00					
Extraversion	0.38***	1.00				
Openness	0.45***	0.45***	1.00			
Neuroticism	–0.19	–0.13	–0.21	1.00		
SR-Negative	−0.28***	−0.21**	−0.35***	0.44**	1.00	
SR-Positive	0.55***	0.42***	0.67***	−0.33**	−0.35***	1.00

*Factor loading: λ, C: Conscientiousness, E: Extraversion, O: Openness, N: Neuroticism, SRN: SR-Negative, SRP: SR-Positive.*

Loadings and correlations with absolute value of ≥0.30 are in boldface. Reversed scored facets are italicized.

*p < 0.10, **p < 0.05, ***p < 0.01.

## Study 2

Another one of the official languages that the SAPI has been translated into is Southern Sotho. Southern Sotho forms part of the Sotho group of languages in South Africa, along with Setswana and Sepedi. People mostly in Lesotho and the Free State speak Southern Sotho (Mesthrie, 2002). The language is spoken by approximately two million people, which creates a demand for psychological assessments in Southern Sotho that can be used in work, community, and counselling contexts (Wissing et al., 2010). Researchers replicated the Five-Factor Model (FFM) within the South African context (Heuchert et al., 2000; Ramsay et al., 2008), and while Ramsay et al. (2008) did obtain construct validity for an FFM assessment among Southern Sotho participants, Heaven and Pretorius (1998) found that the FFM did not replicate well within the Southern Sotho and Sepedi speaking sample. One of the objectives of the overall development of the SAPI was that it would indeed replicate across languages such as Southern Sotho.

### Participants

#### Inclusion and Exclusion

This study focused specifically on Southern Sotho speaking people, 18 years and older, who understood Southern Sotho and who could read the Southern Sotho language.

#### Participant Characteristics

The sample consisted of 42% men and 49% women. Most of the participants were between the ages of 18 and 27 (55%). African people made up 88% of the participants, with only 0.7% of individuals identifying as Coloured. Most of the participants (59%) spoke Southern Sotho as a home language. The majority of the participants had a Grade 12 (46%), while 11% had completed only Grade 9, and 26% had completed a post-school qualification. Sixty-seven percent indicated their ability to read English was good or very good. No multivariate outliers were found in the Southern Sotho data set.

#### Sample Size

The initial data set of 417 was reduced to 293 once the extreme missing values were deleted.

### Results

The Sesotho speaking participants (Table 6) did not at all endorse extreme response styles such as “strongly disagree” or “strongly agree.” Only the Emotional Balance, Hostility-Egoism and Conflict Seeking facets were mainly endorsed on the “disagreed” scale, while the Deceitfulness, Arrogance, and Negative Emotionality facets were frequently endorsed on the “neutral” scale. All of the positively orientated facets were scored on the “agree” scale.

**TABLE 6:** Descriptive statistics of the SAPI-SS facets.

Variable	Mean	Std. Deviation	Alpha	Number of items
**Conscientiousness**				
Achievement Orientation	3.94	0.47	0.68	10
Orderliness	3.87	0.44	0.72	13
Traditionalism-Religiosity	3.95	0.69	0.36	3
Integrity	3.96	0.48	0.76	13
**Extraversion**				
Playfulness	3.75	0.54	0.47	6
Sociability	3.70	0.65	0.52	5
**Openness**				
Broad-Mindedness	3.84	0.60	0.56	5
Epistemic Curiosity	4.01	0.52	0.54	6
Intellect	3.81	0.47	0.67	10
**Neuroticism**				
Emotional Balance	2.11	0.48	0.47	6
Negative Emotionality	2.85	0.61	0.62	8
**SR-Negative**				
Arrogance	2.73	0.81	0.70	6
Conflict Seeking	2.45	0.77	0.75	7
Deceitfulness	2.68	0.82	0.79	7
Hostility Egoism	2.43	0.68	0.83	14
**SR-Positive**				
Integrity*	3.96	0.48	0.76	13
Empathy	3.83	0.52	0.53	6
Facilitation	3.80	0.51	0.69	9
Interpersonal Relatedness	3.83	0.53	0.70	8
Social Intelligence	3.91	0.55	0.40	4
Warm Heartedness	3.91	0.46	0.72	11

*The Integrity facet forms part of both the Conscientiousness and SR-Positive factors.*

Thirteen items were identified and removed that decreased certain facets’ reliability and displayed poor model fit. Most of the facet reliabilities ranged between 0.52 and 0.83 (*M* = 0.68). Four facets had alphas > 0.50; however, the Playfulness, Emotional Balance, and Social Intelligence facet displayed acceptable to excellent model fit and was retained for subsequent analyses. For more information regarding the item loadings, please see Appendix A. Whereas the Traditionalism-Religiosity facet, as within the Tshivenda version, displayed poor internal consistency (α = 0.36) and model fit that could not be assessed. Consequently, the facet was removed from further analyses.

The ESEM solution for the SAPI-SS generated good fit to the data (χ^2^ = 149.109; *df* = 72; CFI = 0.97; TLI = 0.94; RMSEA = 0.06; 90% CI for the RMSEA = 0.05–0.07; SRMR = .02). Table 7 indicates adequate factor loadings for the facets that loaded on their expected factors, ranging between 0.47 and 0.88 (*M* = 0.60). Cross-loadings ranged between −0.54 and 0.51 (*M* = 0.04); however, none of the cross-loadings loaded better on alternative factors. The Neuroticism factor produced problematic cross-loadings since both its facets had poor factor loadings on Neuroticism. Emotional Balance loaded adequately on the Conscientiousness factor (λ = 0.39), while Negative Emotionality loaded strongly on the SR-Negative factor (λ = –0.54). The definitions of Conscientiousness and SR-Negative could therefore be altered, given the addition of Emotional Balance and Negative Emotionality, respectively. As was estimated, Integrity had acceptable factor loadings on both Conscientiousness and SR-Positive. While Neuroticism did not correlate strongly with any of the remaining factors (due to its poor factor representation), strong moderate correlations were present, especially between SR-Positive and Openness (*r* = 0.65).

**TABLE 7:** Standardized estimates of the SAPI-SS based on the ESEM solution.

Variable	λ					
	C	E	O	N	SRN	SRP
**Conscientiousness**						
Achievement Orientation	0.47***	–0.02	**0.34**	0.07	–0.02	0.08
Orderliness	0.68***	0.10	0.16**	0.00	–0.05	0.04
Integrity	0.51***	0.07	0.00	0.06	−0.23***	0.32***
**Extraversion**						
Playfulness	–0.16	0.42**	0.36**	–0.01	0.20	0.08
Sociability	0.05	**0.69**	−0.15*	0.07	0.08	0.19
**Openness**						
Broad-Mindedness	0.05	0.15	**0.67**	–0.13	–0.11	0.02
Epistemic Curiosity	0.11	0.01	**0.43**	–0.15	–0.14	0.23
Intellect	0.27	0.04	**0.41**	0.42*	0.19	0.15
**Neuroticism**						
*Emotional Balance*	0.39***	–0.03	0.25*	0.04	–0.07	0.19
Negative Emotionality	–0.02	0.23	0.01	0.22	−0.54***	−0.31**
**SR-Negative**						
Arrogance	0.26***	0.09	–0.09	–0.22	0.78***	−0.20*
Conflict Seeking	−0.23***	0.06	0.01	0.03	0.79***	0.03
Deceitfulness	−0.12*	–0.05	0.03	0.02	0.83***	–0.01
Hostility Egoism	−0.08*	0.00	–0.04	0.01	0.88***	–0.07
**SR-Positive**						
Integrity	0.51***	0.07	0.00	0.06	−0.23***	0.32***
Empathy	−0.22*	–0.05	0.11	–0.04	–0.10	0.81***
Facilitation	0.26***	0.10	0.12	0.08	–0.03	0.39**
Interpersonal Relatedness	0.14	0.22**	0.06	0.04	–0.03	0.52***
Social Intelligence	0.07	0.17	0.14	–0.15	–0.13	0.45***
Warm Heartedness	0.14	0.09	–0.08	0.01	–0.01	0.75**
**Factor correlations**						
Conscientiousness	1.00					
Extraversion	0.35**	1.00				
Openness	0.50**	0.36*	1.00			
Neuroticism	0.21	0.07	0.09	1.00		
SR-Negative	−**0.31**	–0.21	–0.16	–0.01	1.00	
SR-Positive	0.55***	**0.50**	0.65***	0.16	–0.18	1.00

*Factor loading: λ, C: Conscientiousness, E: Extraversion, O: Openness, N: Neuroticism, SRN: SR-Negative, SRP: SR-Positive.*

Loadings and correlations with absolute values of ≥0.30 are in boldface. Reversed scored facets are italicized.

*p < 0.10, **p < 0.05, ***p < 0.01.

## Discussion

South African research in personality assessment has frequently highlighted the challenges of using imported personality measures when evaluating South Africans from diverse cultures and ethnicities. The SAPI seems to bridge this gap by presenting an instrument that has been developed using an emic-etic approach, as well as having the assessment available in all 11 official languages. The various language versions have, however, not been psychometrically evaluated to determine whether they replicate the original SAPI-EN factor structure as offered by Fetvadjiev et al. (2015). The aim of the current study was, therefore, to examine the factor structures of the Tshivenda (SAPI-VE) and the Southern Sotho (SAPI-SS) versions of the SAPI.

Both language groups revealed a lack of extreme response styles (ERS; e.g., “strongly disagree,” “strongly agree”) and rather acquiesced when answering different versions of the SAPI. Contrary to these findings, Batchelor and Miao (2016) found in a meta-analysis of ERS that Africans generally choose ERS when answering questionnaires. However, these studies only included African American participants outside of Africa (Hui and Triandis, 1989; Johnson et al., 2005; Danner et al., 2015; He and van de Vijver, 2017; Rammstedt et al., 2017). As Eaton and Louw (2000) (p. 211) stated, “… this continent [Africa] has been ignored almost entirely” when investigating psychological assessments. Harzing (2006) found that Hofstede’s cultural dimensions of a country influence the response styles of participants from that country. A cultural group’s response style, be it ERS or acquiescence, may be an expression of how that culture communicates (Smith, 2004). Applying Hofstede’s cultural dimensions, the Tshivenda and Sesotho cultures can be considered as high power distance and collectivistic cultures (Darley and Blankson, 2008). Therefore, the Tshivenda and Sesotho participants may have selected a middling response style to adhere to group consensus and accentuating relational harmony, as opposed to expressing strong individual opinions (Johnson et al., 2005). He and van de Vijver (2017) cautioned that differences in culture might influence how individuals score their assessments, i.e., instrument bias. However, the current study’s results are supported by Morton et al. (2018, 2019), according to which the same response style was found for the SAPI-EN across the different ethnic groups.

The Traditionalism-Religiosity facet proved to be problematic in both language versions, generating internal consistency and model fit that could not be assessed. Traditionalism-Religiosity with the SAPI model refers to behaviour in which a person displays respect towards one’s own culture, as well as being religious. According to Valchev et al. (2013), subfacets dealing with Traditionalism and Religiosity were mentioned more often by Blacks than Whites; confirming conclusions by Harkness and Super (1977) that “Obedience and respect are required in many relationships between people of differing status in sub-Saharan Africa” (p. 329). The facet in its current form, however, only contains four items; two relating to traditionalism and two relating to religiosity. The small number of items most likely played a role in the poor reliability coefficients. Fetvadjiev et al. (2015) similarly reported mediocre reliability coefficients (α = 0.57) for the Traditionalism-Religiosity (SAPI-EN) facet for African participants, although the factor loadings for the African sample were acceptable (λ = 0.56). A possible conceptual concern would be the inclusion of two seemingly distinct aspects of personality in one facet. It can be argued that traditionalism and religiosity may be related but should be considered as separate entities. Traditionalism seems to generally represent the preserving of, and adherence to established order, doctrines and customs passed on by generations within a specific culture, while religiosity denotes the observance of practices and beliefs devoted to a supreme divinity or reality (Merriam-Webster, 2020a,b). While both behaviours are connected through the preservation of order and beliefs, the entity from which these behaviours originate and are directed at differs. Morton et al. (2019) similarly found in their study that the factor loading of the SAPI-EN Traditionalism-Religiosity facet displayed a poor factor loading (λ = 0.26) among a general South African population. Therefore, using the quantitative evidence as well as conceptual arguments, a few suggestions for future use: (a) remove the facet in its entirety from the SAPI; (b) expand the number of items for the facet, or (3) divide the two sections of the facet and add additional items to each facet.

The results indicated that the ESEM model provided a good model fit in both language versions of the SAPI. The factor correlation results of both studies indicated that the SAPI factors could be regarded as six separate factors. A few noteworthy cross-loadings are discussed below.

The Neuroticism factor was problematic in both studies. Overall, the SAPI Neuroticism factor measures to what extent a person can control their emotions by being calm and confident and avoid being thoughtless and rash. The Emotional Balance facet specifically refers to behaviour in which people act with self-control in challenging situations and when people acknowledge their own emotions, having consideration and understanding for said emotions. People who score high on the Negative Emotionality factor generally display traits of being indignant, apprehensive, nervous, and fearful.

Within the SAPI-VE study, Emotional Balance did not load sufficiently on any of the factors, while Negative Emotionality had a very strong factor loading on the Neuroticism factor, albeit borderline significantly. In her study on the Tshivenda language group’s implicit perspectives on personality, Ntsieni (2006) identified 150 personality characteristics that were grouped into eight categories: Conscientiousness, dominance, emotionality, intellect, interpersonal relatedness, meanness, sociability, and other. The description of the emotionality category in this instance encompasses both content from the SAPI’s Emotional Balance and Negative Emotionality facets. Given that these facets are well-represented within the Tshivenda language group, it is an anomaly as to why the translated Emotional Balance facet did not produce a strong factor loading on any of the SAPI factors. It is, therefore, speculated that the problem does not lie in the content of the items but rather in the translation thereof. As such, this facet’s translations should be revisited.

The results for the SAPI-SS indicated that the two Neuroticism facets should rather be included in the Conscientiousness and SR-Negative factors. Kruger (2006) studied the embedded perspectives of personality among Sesotho speaking people and isolated 94 personality characteristics which are represented by seven personality categories: Conscientiousness, Dominance, Emotionality, Interpersonal Relatedness, Meanness, Sociability, and Other. These categories are similar to those of Ntsieni (2006). However, the delineation of the various categories differed across the two language groups. The Sesotho description of emotionality differed from Tshivenda’s portrayal in that the Sesotho perspective of emotionality encapsulates behaviour that is more related to the SR-Negative factor, such as argumentativeness and arrogance. Both the Emotional Balance facet and the Conscientiousness factor include an aspect of control. Conscientiousness incorporates aspects of achieving goals, being thorough, organized and consistent, which point to behaviour that helps a person maintain control over life and where they want to go. Similarly, a person who scores high on Emotional Balance tends to maintain self-control in problematic circumstances. Kruger (2006) found that Sesotho people consider having control over self as part of being conscientious. It is plausible that the Negative Emotionality facet would fall under the SR-Negative factor and the Emotional Balance facet load on the Conscientiousness factor.

These results are in contrast with Fetvadjiev et al. (2015) and Morton et al. (2019), who provided evidence that both Emotional Balance and Negative Emotionality had adequate to strong factor loadings on the Neuroticism factor, regardless of race. However, Fetvadjiev et al. (2015) did find that the Negative Emotionality facet also generated an acceptable (cross) loading on the SR-Negative factor among a sample of Black participants, which correlates with the suggestion within the SAPI-SS results that Negative Emotionality could load under the SR-Negative factor.

For the SAPI-VE, Achievement Orientation generated a stronger factor loading on the Openness factor, while for the SAPI-SS Achievement Orientation loaded sufficiently on the Conscientiousness factor but still produced cross-loading on the Openness factor. These findings are supported to some extent by the results of Fetvadjiev et al. (2015), of which the factor structure of the SAPI-EN also showed that the Achievement Orientation facet also loaded on both the Conscientiousness and Openness factors. Examining the structure replicability across different demographic groups, Fetvadjiev et al. (2015) found lower replicability across the Conscientiousness and Openness factors; merging these factors seemed to improve replicability. However, they opted to keep the two factors separate, given theoretical considerations as well as the moderate correlation between the two factors. Given that Achievement Orientation in the SAPI-VE did not have a meaningful loading on Conscientiousness, it suggests that the translations of the items should be revisited to ensure that the meaning of the constructs between the various language versions remains equivalent.

The current studies were not without limitations; however, these limitations serve as key lessons to be attended to in future studies. While the researchers initially gathered over 400 participants for each study, both studies yielded just under 300 usable questionnaires. Larger sample sizes may have generated more meaningful results, as larger sample sizes increase the precision in the given data (Biau et al., 2008). Second, participants were gathered only in areas around Gauteng. Perhaps targeting other areas where Tshivenda/Sesotho people reside would have created more significant variation in the results, as the sample size may have been more representative of the population (Biau et al., 2008). A limitation of Study 2 was that the sample included individuals who spoke Southern Sotho and was not limited only to individuals who spoke Southern Sotho as a home language and identified as ethnically Southern Sotho.

It is suggested that future research administer the native-language versions of the SAPI in conjunction with the SAPI-EN to the same participants; this may assist in examining whether the language translations influence the structure of the SAPI. An investigation of whether acquiescence will be decreased by increasing or decreasing response categories is also warranted. A more detailed analysis at the item level is also needed, especially a comparison between the various SAPI versions. It will also be valuable to investigate the factor structures of the SAPI among the other eight official languages to determine whether the current studies’ results are a unique or expected phenomenon in terms of the SAPI.

## Conclusion

According to Tanzer (2005), the process of developing a valid multicultural or multilingual measurement will comprise more than merely rewriting, reworking, or adapting text from a source language to a target language. Indeed, as Rogers et al. (2003) found in their research review, tests translated from a source language to a target language frequently do not yield equivalent constructs. The results of this study show that, despite the universal characteristics of the SAPI in terms of the 11 language groups, specific patterns emerged in the Tshivenda and Sesotho versions of the SAPI that are to some extent justifiable based on the initially identified implicit perspectives of personality of the two language groups. While the overall fit of the six-factor model was excellent, it seems that the SAPI-VE and SAPI-SS cannot yet be positioned as equitable alternatives when using an indigenous version of the SAPI is needed. However, even though the results were not in line with initial expectations, the current study can still be seen as pioneering and a step in the right direction to ensure that the necessary cultural sensitivity and rigor are applied in developing an indigenous personality inventory that can be validly and reliably administered in different languages. As such, the SAPI project provides “…a useful blueprint of the combined emic-etic approach to ensure comprehensive coverage of the psychological constructs and their cultural relevance to the local context” (Cheung et al., 2011, p. 600).

## Data Availability Statement

The datasets generated during and analysed during the current study are not publicly available due to the copyrighted items. Requests to access the datasets should be directed to chill@uj.ac.za.

## Ethics Statement

The studies involving human participants were reviewed and approved by the University of Johannesburg Departmental Ethical Committee. Ethical clearance numbers: IPPM2017/061(M) and IPPM2017/101(M). The study involved human participants and all procedures were in accordance with the ethical standards set out by the institutional research committee, as well as the Rules of Conduct Pertaining Specifically to Psychology as set out by the Health Professions Council of South Africa’s Professional Board of Psychology (Form 223). The patients/participants provided their written informed consent to participate in this study.

## Author Contributions

CH, MH, and LL contributed equally to this article.

## Conflict of Interest

The authors declare that the research was conducted in the absence of any commercial or financial relationships that could be construed as a potential conflict of interest.

## Footnotes

1 “The Coloured population have a mixed lineage, which often comprises the indigenous Khoisan genes combined with African slaves that were brought here from all over the continent, and white settlers” (SA-Venues, 2020).

2 Researchers interested in the SAPI project or the scale for research purposes, please email CH (chill@uj.ac.za) or Alewyn Nel (alewyn.nel@up.ac.za).
