# Supplementary material for: Validating Indigenous Versions of the South African Personality Inventory
Source: Front Psychol. 2021 May 20;12:556565. doi: 10.3389/fpsyg.2021.556565 (PMC8172579; doi:10.3389/fpsyg.2021.556565)
Supplement: Supplementary file 1 [file Data_Sheet_1.docx]

Appendix A

| **Facets** | **λ** | ***p*** |  | **λ** | ***p*** |
| --- | --- | --- | --- | --- | --- |
|  | **SAPI-SS** | |  | **SAPI-V** | |
| **Conscientiousness** | | | | | |
| *Achievement Orientation* | | | | | |
| C-AO item 1 | 0.255 | 0.000 |  | 0.274 | 0.001 |
| C-AO item 2 | 0.379 | 0.000 |  | 0.214 | 0.003 |
| C-AO item 3 | 0.289 | 0.001 |  | 0.361 | 0.000 |
| C-AO item 4 | 0.367 | 0.000 |  | 0.615 | 0.000 |
| C-AO item 5 | 0.350 | 0.000 |  | 0.398 | 0.000 |
| C-AO item 6 | - | - |  | 0.639 | 0.000 |
| C-AO item 7 | 0.344 | 0.000 |  | 0.422 | 0.000 |
| C-AO item 8 | 0.557 | 0.000 |  | 0.700 | 0.000 |
| C-AO item 9 | 0.478 | 0.000 |  | 0.492 | 0.000 |
| C-AO item 10 | 0.550 | 0.000 |  | 0.465 | 0.000 |
| C-AO item 11 | 0.581 | 0.000 |  | 0.300 | 0.000 |
| *Integrity* |  |  |  |  |  |
| CSRP-I item 1 | 0.366 | 0.000 |  | 0.477 | 0.000 |
| CSRP-I item 2 | 0.440 | 0.000 |  | 0.482 | 0.000 |
| CSRP-I item 3 | 0.491 | 0.000 |  | 0.209 | 0.003 |
| CSRP-I item 4 | 0.561 | 0.000 |  | 0.467 | 0.000 |
| CSRP-I item 5 | 0.410 | 0.000 |  | 0.344 | 0.000 |
| CSRP-I item 6 | 0.365 | 0.000 |  | 0.648 | 0.000 |
| CSRP-I item 7 | 0.429 | 0.000 |  | 0.479 | 0.000 |
| CSRP-I item 8 | 0.546 | 0.000 |  | 0.389 | 0.000 |
| CSRP-I item 9 | 0.571 | 0.000 |  | 0.313 | 0.000 |
| CSRP-I item 10 | 0.439 | 0.000 |  | 0.483 | 0.000 |
| CSRP-I item 11 | 0.482 | 0.000 |  | 0.433 | 0.000 |
| CSRP-I item 12 | 0.432 | 0.000 |  | 0.386 | 0.000 |
| CSRP-I item 13 | 0.196 | 0.009 |  | 0.528 | 0.000 |
| *Orderliness* |  |  |  |  |  |
| C-O item 1 | 0.350 | 0.000 |  | 0.295 | 0.000 |
| C-O item 2 | 0.258 | 0.002 |  | 0.247 | 0.000 |
| C-O item 3 | 0.253 | 0.000 |  | 0.325 | 0.000 |
| C-O item 4 | 0.421 | 0.000 |  | 0.491 | 0.000 |
| C-O item 5 | 0.316 | 0.000 |  | 0.554 | 0.000 |
| C-O item 6 | 0.483 | 0.000 |  | 0.384 | 0.000 |
| C-O item 7 | 0.533 | 0.000 |  | 0.583 | 0.000 |
| C-O item 8 | 0.627 | 0.000 |  | 0.560 | 0.000 |
| C-O item 9 | 0.357 | 0.000 |  | 0.588 | 0.000 |
| C-O item 10 | 0.512 | 0.000 |  | 0.453 | 0.000 |
| C-O item 11 | 0.404 | 0.000 |  | 0.518 | 0.000 |
| C-O item 12 | 0.541 | 0.000 |  | 0.487 | 0.000 |
| C-O item 13 | 0.212 | 0.007 |  | 0.438 | 0.000 |
| *Traditionalism–Religiosity* | | | | | |
| C-TR item 1 | - | - |  | 0.680 | 0.001 |
| C-TR item 2 | 0.285 | 0.031 |  | - | - |
| C-TR item 3 | 0.284 | 0.025 |  | 0.604 | 0.001 |
| C-TR item 4 | 0.713 | 0.012 |  | 0.188 | 0.025 |
| **Extraversion** | | | | | |
| *Playfulness* |  |  |  |  |  |
| EX-P item 1 | 0.202 | 0.032 |  | 0.370 | 0.000 |
| EX-P item 2 | 0.435 | 0.000 |  | 0.411 | 0.000 |
| EX-P item 3 | 0.377 | 0.000 |  | 0.404 | 0.000 |
| EX-P item 4 | 0.548 | 0.000 |  | 0.669 | 0.000 |
| EX-P item 5 | 0.362 | 0.001 |  | 0.448 | 0.000 |
| EX-P item 6 | 0.231 | 0.052 |  | 0.284 | 0.001 |
| *Sociability* |  |  |  |  |  |
| EX-S item 1 | - | - |  | - | - |
| EX-S item 2 | - | - |  | 0.463 | 0.000 |
| EX-S item 3 | 0.402 | 0.000 |  | 0.298 | 0.001 |
| EX-S item 4 | 0.456 | 0.000 |  | 0.342 | 0.000 |
| EX-S item 5 | 0.693 | 0.000 |  | 0.779 | 0.000 |
| EX-S item 6 | 0.269 | 0.002 |  | 0.479 | 0.000 |
| EX-S item 7 | 0.328 | 0.000 |  | 0.681 | 0.000 |
| **Neuroticism** | | | | | |
| *Emotional Balance* |  |  |  |  |  |
| N-EB item 1 | 0.255 | 0.009 |  | 0.207 | 0.018 |
| N-EB item 2 | 0.253 | 0.021 |  | 0.397 | 0.000 |
| N-EB item 3 | 0.303 | 0.001 |  | 0.714 | 0.000 |
| N-EB item 4 | 0.555 | 0.000 |  | 0.384 | 0.000 |
| N-EB item 5 | 0.297 | 0.007 |  | 0.519 | 0.000 |
| N-EB item 6 | - | - |  | 0.382 | 0.000 |
| N-EB item 7 | 0.468 | 0.000 |  | 0.377 | 0.000 |
| *Negative Emotionality* |  |  |  |  |  |
| N-NE item 1 | 0.502 | 0.000 |  | 0.480 | 0.000 |
| N-NE item 2 | 0.251 | 0.003 |  | - | - |
| N-NE item 3 | 0.385 | 0.000 |  | 0.429 | 0.000 |
| N-NE item 4 | 0.349 | 0.000 |  | 0.299 | 0.000 |
| N-NE item 5 | - | - |  | 0.297 | 0.000 |
| N-NE item 6 | 0.413 | 0.000 |  | 0.632 | 0.000 |
| N-NE item 7 | - | - |  | - | - |
| N-NE item 8 | 0.483 | 0.000 |  | 0.462 | 0.000 |
| N-NE item 9 | 0.432 | 0.000 |  | 0.380 | 0.000 |
| N-NE item 10 | 0.476 | 0.000 |  | 0.675 | 0.000 |
| **Openness** | | | | | |
| *Broad-Mindedness* |  |  |  |  |  |
| OP-BM item 1 | 0.278 | 0.012 |  | 0.077 | 0.000 |
| OP-BM item 2 | - | - |  | 0.082 | 0.000 |
| OP-BM item 3 | 0.262 | 0.017 |  | 0.065 | 0.000 |
| OP-BM item 4 | 0.278 | 0.010 |  | 0.087 | 0.000 |
| OP-BM item 5 | 0.730 | 0.000 |  | 0.093 | 0.000 |
| OP-BM item 6 | 0.594 | 0.000 |  | 0.082 | 0.000 |
| *Epistemic Curiosity* |  |  |  |  |  |
| OP-EC item 1 | 0.271 | 0.000 |  | - | - |
| OP-EC item 2 | 0.310 | 0.000 |  | 0.281 | 0.000 |
| OP-EC item 3 | 0.369 | 0.000 |  | 0.453 | 0.000 |
| OP-EC item 4 | 0.746 | 0.000 |  | 0.624 | 0.000 |
| OP-EC item 5 | 0.287 | 0.004 |  | 0.496 | 0.000 |
| OP-EC item 6 | 0.552 | 0.000 |  | 0.392 | 0.000 |
| *Intellect* |  |  |  |  |  |
| OP-I item 1 | 0.357 | 0.000 |  | 0.455 | 0.000 |
| OP-I item 2 | 0.399 | 0.000 |  | 0.432 | 0.000 |
| OP-I item 3 | 0.448 | 0.000 |  | 0.484 | 0.000 |
| OP-I item 4 | 0.431 | 0.000 |  | 0.475 | 0.000 |
| OP-I item 5 | 0.507 | 0.000 |  | 0.590 | 0.000 |
| OP-I item 6 | 0.248 | 0.001 |  | 0.389 | 0.000 |
| OP-I item 7 | 0.479 | 0.000 |  | 0.448 | 0.000 |
| OP-I item 8 | 0.405 | 0.000 |  | 0.420 | 0.000 |
| OP-I item 9 | 0.356 | 0.000 |  | 0.361 | 0.000 |
| OP-I item 10 | - | - |  | - | - |
| OP-I item 11 | 0.497 | 0.000 |  | 0.323 | 0.000 |
| **Social-Relational Negative** | | | | | |
| *Arrogance* |  |  |  |  |  |
| SRN-A item 1 | 0.575 | 0.000 |  | 0.572 | 0.000 |
| SRN-A item 2 | 0.349 | 0.000 |  | - | - |
| SRN-A item 3 | 0.683 | 0.000 |  | 0.446 | 0.000 |
| SRN-A item 4 | 0.469 | 0.000 |  | 0.665 | 0.000 |
| SRN-A item 5 | 0.576 | 0.000 |  | 0.616 | 0.000 |
| SRN-A item 6 | 0.568 | 0.000 |  | 0.529 | 0.000 |
| *Conflict-Seeking* |  |  |  |  |  |
| SRN-CS item 1 | 0.387 | 0.000 |  | 0.675 | 0.000 |
| SRN-CS item 2 | 0.603 | 0.000 |  | 0.474 | 0.000 |
| SRN-CS item 3 | 0.638 | 0.000 |  | - | - |
| SRN-CS item 4 | 0.467 | 0.000 |  | - | - |
| SRN-CS item 5 | 0.530 | 0.000 |  | 0.412 | 0.000 |
| SRN-CS item 6 | 0.665 | 0.000 |  | 0.552 | 0.000 |
| SRN-CS item 7 | 0.527 | 0.000 |  | 0.496 | 0.000 |
| *Deceitfulness* |  |  |  |  |  |
| SRN-D item 1 | 0.599 | 0.000 |  | 0.492 | 0.000 |
| SRN-D item 2 | 0.472 | 0.000 |  | 0.551 | 0.000 |
| SRN-D item 3 | 0.670 | 0.000 |  | 0.375 | 0.000 |
| SRN-D item 4 | 0.755 | 0.000 |  | 0.224 | 0.001 |
| SRN-D item 5 | 0.610 | 0.000 |  | 0.742 | 0.000 |
| SRN-D item 6 | 0.487 | 0.000 |  | 0.527 | 0.000 |
| SRN-D item 7 | 0.551 | 0.000 |  | 0.654 | 0.000 |
| *Hostility–Egoism* |  |  |  |  |  |
| SRN-HE item 1 | 0.326 | 0.000 |  | 0.163 | 0.031 |
| SRN-HE item 2 | 0.604 | 0.000 |  | 0.396 | 0.000 |
| SRN-HE item 3 | 0.622 | 0.000 |  | 0.280 | 0.001 |
| SRN-HE item 4 | 0.272 | 0.000 |  | 0.354 | 0.000 |
| SRN-HE item 5 | 0.559 | 0.000 |  | 0.338 | 0.000 |
| SRN-HE item 6 | 0.412 | 0.000 |  | 0.434 | 0.000 |
| SRN-HE item 7 | 0.491 | 0.000 |  | 0.412 | 0.000 |
| SRN-HE item 8 | 0.597 | 0.000 |  | 0.532 | 0.000 |
| SRN-HE item 9 | 0.451 | 0.000 |  | 0.508 | 0.000 |
| SRN-HE item 10 | 0.655 | 0.000 |  | 0.508 | 0.000 |
| SRN-HE item 11 | 0.573 | 0.000 |  | 0.527 | 0.000 |
| SRN-HE item 12 | 0.575 | 0.000 |  | 0.461 | 0.000 |
| SRN-HE item 13 | 0.462 | 0.000 |  | - | - |
| SRN-HE item 14 | 0.501 | 0.000 |  | 0.414 | 0.000 |
| **Social-Relational Negative** | | | | | |
| *Empathy* |  |  |  |  |  |
| SRP-E item 1 | 0.288 | 0.001 |  | 0.427 | 0.000 |
| SRP-E item 2 | - | - |  | 0.325 | 0.000 |
| SRP-E item 3 | 0.423 | 0.000 |  | 0.368 | 0.000 |
| SRP-E item 4 | 0.589 | 0.000 |  | 0.439 | 0.000 |
| SRP-E item 5 | 0.450 | 0.000 |  | 0.507 | 0.000 |
| SRP-E item 6 | 0.532 | 0.000 |  | 0.509 | 0.000 |
| SRP-E item 7 | 0.570 | 0.000 |  | 0.540 | 0.000 |
| *Facilitating* |  |  |  |  |  |
| SRP-F item 1 | 0.534 | 0.000 |  | 0.362 | 0.000 |
| SRP-F item 2 | 0.032 | 0.672 |  | 0.519 | 0.000 |
| SRP-F item 3 | 0.262 | 0.002 |  | 0.624 | 0.000 |
| SRP-F item 4 | 0.544 | 0.000 |  | 0.459 | 0.000 |
| SRP-F item 5 | 0.329 | 0.000 |  | 0.619 | 0.000 |
| SRP-F item 6 | 0.279 | 0.001 |  | 0.482 | 0.000 |
| SRP-F item 7 | 0.380 | 0.000 |  | 0.428 | 0.000 |
| SRP-F item 8 | 0.297 | 0.001 |  | 0.523 | 0.000 |
| SRP-F item 9 | 0.432 | 0.000 |  | 0.570 | 0.000 |
| SRP-F item 10 | 0.314 | 0.000 |  | 0.540 | 0.000 |
| *Interpersonal Relatedness* | | | | | |
| SRP-IR item 1 | 0.254 | 0.001 |  | 0.295 | 0.000 |
| SRP-IR item 2 | 0.615 | 0.000 |  | 0.367 | 0.000 |
| SRP-IR item 3 | 0.538 | 0.000 |  | 0.559 | 0.000 |
| SRP-IR item 4 | 0.543 | 0.000 |  | 0.437 | 0.000 |
| SRP-IR item 5 | 0.427 | 0.000 |  | 0.459 | 0.000 |
| SRP-IR item 6 | 0.487 | 0.000 |  | 0.583 | 0.000 |
| SRP-IR item 7 | 0.494 | 0.000 |  | 0.720 | 0.000 |
| SRP-IR item 8 | 0.460 | 0.000 |  | 0.435 | 0.000 |
| *Social Intelligence* |  |  |  |  |  |
| SRP-SI item 1 | 0.344 | 0.000 |  | 0.380 | 0.000 |
| SRP-SI item 2 | 0.239 | 0.016 |  | 0.670 | 0.000 |
| SRP-SI item 3 | 0.449 | 0.000 |  | 0.520 | 0.000 |
| SRP-SI item 4 | 0.554 | 0.000 |  | 0.717 | 0.000 |
| *Warm-Heartedness* |  |  |  |  |  |
| SRP-WH item 1 | 0.376 | 0.000 |  | 0.681 | 0.000 |
| SRP-WH item 2 | 0.503 | 0.000 |  | 0.371 | 0.000 |
| SRP-WH item 3 | 0.564 | 0.000 |  | 0.484 | 0.000 |
| SRP-WH item 4 | 0.357 | 0.000 |  | 0.372 | 0.000 |
| SRP-WH item 5 | 0.474 | 0.000 |  | 0.349 | 0.000 |
| SRP-WH item 6 | 0.492 | 0.000 |  | 0.539 | 0.000 |
| SRP-WH item 7 | 0.541 | 0.000 |  | 0.371 | 0.000 |
| SRP-WH item 8 | 0.389 | 0.000 |  | 0.389 | 0.000 |
| SRP-WH item 9 | 0.363 | 0.000 |  | 0.347 | 0.000 |
| SRP-WH item 10 | 0.406 | 0.000 |  | 0.322 | 0.000 |
| SRP-WH item 11 | 0.354 | 0.000 |  | 0.335 | 0.000 |

*Note.* (-): item decreased the facet’s reliability significantly and was omitted from analysis.
